# Isolation, culturing and gene expression profiling of inner mass cells from stable and vulnerable carotid atherosclerotic plaques

**DOI:** 10.1371/journal.pone.0218892

**Published:** 2019-06-26

**Authors:** Olga A. Novikova, Zhanna K. Nazarkina, Anna V. Cherepanova, Petr P. Laktionov, Boris P. Chelobanov, Ivan S. Murashov, Roman V. Deev, Evgeny A. Pokushalov, Andrey A. Karpenko, Pavel P. Laktionov

**Affiliations:** 1 “E. Meshalkin National Medical Research Center”, Ministry of Health of the Russian Federation, Novosibirsk, Russia; 2 Laboratory of Molecular Medicine, SB RAS Institute of Chemical Biology and Fundamental Medicine, Novosibirsk, Russia; 3 Laboratory of Genomics, SB RAS Institute of Molecular and Cellular Biology, Novosibirsk, Russia; 4 Novosibirsk State University, Novosibirsk, Russia; 5 Ryazan State Medical University, Ryazan, Russia; Monash University, AUSTRALIA

## Abstract

The connective tissue components that form the atherosclerotic plaque body are produced by the plaque inner mass cells (PIMC), located inside the plaque. We report an approach to isolate and culture cells from the connective tissue of stable and vulnerable human atherosclerotic plaques based on elimination of non-connective tissue cells such as blood and non-plaque intima cells with a lysis buffer. The resulting plaque cells were characterized by growth capacity, morphology, transcriptome profiling and specific protein expression. Plaque cells slowly proliferated for up to three passages unaffected by the use of proliferation stimulants or changes of culture media composition. Stable plaques yielded more cells than vulnerable ones. Plaque cell cultures also contained several morphological cellular types. RNA-seq profiles of plaque cells were different from any of the cell types known to be involved in atherogenesis. The expression of the following proteins was observed in cultured plaque cells: smooth muscle cells marker α-SMA, macrophage marker CD14, extracellular matrix proteins aggrecan, fibronectin, neovascularisation markers VEGF-A, CD105, cellular adhesion receptor CD31 and progenitor/dedifferentiation receptor CD34. Differential expression of several notable transcripts in cells from stable and vulnerable plaques suggests the value of plaque cell culture studies for the search of plaque vulnerability markers.

## Introduction

Atherosclerosis is a chronic complex disease of the arterial wall involving several pathological processes which can vary between patients [1]. Several cell types are involved in atherogenesis: endothelial cells, vascular smooth muscle cells (VSMC), monocyte-derived macrophages and other inflammatory cells, including T cells, B cells, and dendritic cells [2].

An atherosclerotic plaque is strongly influenced by the surrounding and circulating cells (through direct contacts as well as cytokines and growth factors) [3] and mechanical forces [4]. However, the formation of plaque stroma, neovascularisation and thrombogenesis [5], calcification [6] and other key structural processes are presumably driven by the plaque inner mass cells (PIMC) comprised mainly of connective tissue cells entombed in the plaque, including cells specific to atherosclerotic plaque compartments such as areas of calcification.

These cells are responsible for extracellular matrix proteins (ECM) production, fibrous cap formation, hydroxyapatite deposition and respond to the signals from surrounding cells, thus being the key actors of atherogenesis and determining plaque phenotype (stable plaque (s-plaque) with thick fibrous cap versus prone to rupture vulnerable plaque (v-plaque) with thinning fibrous cap and heightened inflammatory state) [1].

A study of human PIMC cultures may expand our knowledge about the growth and development of atheromas and contribute to the *in vitro* study of the plaque vulnerability and the search for the new markers of v-plaques. The quantity of PIMC in a plaque is relatively low due to the slow growth and the dense structure of atherosclerotic plaques [7]. Several groups have previously cultured plaque cells after isolation from minced plaque tissue using long-term enzymatic digestion [8–10]. According to these reports, plaque cells in culture resemble smooth muscle cells, although cellular composition of cell cultures derived from plaques may be more heterogeneous. Additionally, plaque surface irregularity and elevated expression of chemokines and adhesion molecules may promote extensive blood cells adhesion [11]. Some of these cells may be retained in plaque cell cultures where they proliferate and/or secrete interleukins or growth factors thus affecting the growth of PIMC [12]. Residual non-plaque intimal cells usually remaining on atheromas after the carotid endarterectomy (CEA) may also proliferate and eventually overgrow PIMC. Different properties of these cells and variations in plaque treatment protocols can significantly affect the composition, phenotype and characteristics of cells in plaque cell cultures.

In this study we have developed a protocol for PIMC isolation which includes an additional step of plaque treatment with lysis buffer to remove non-plaque cells. We have cultured cells from stable and vulnerable plaques (s-PIMC and v-PIMC) up to three passages and characterized their RNA expression profile and the expression of several ECM proteins and markers of atherosclerosis. PIMC cultures may prove to be useful *in vitro* model of cellular interactions in atherosclerotic plaques.

## Materials and methods

### Patients and specimen

The study included a total of 91 patients (Table 1) undergoing CEA for occlusive artery disease. Of these, 24 patients were symptomatic and had an ischaemic event (transient ischemic attack/stroke/amaurosis fugax) within the 6-month period prior to CEA. The investigation was approved by the Local Ethical Committee of the E. Meshalkin National medical investigation center (№42, 17.10.2014). Written informed consent was obtained from all participants according to the Declaration of Helsinki.

**Table 1:** Patient characteristics and plaque vulnerability.

Variable	Plaque vulnerability		p value
	Stable (n = 35) N(%)	Vulnerable (n = 56)N(%)	
Age, years	67 [63; 69]	65.5 [60; 70.5]	0.91
Male sex, % (n)	20 (57.1%)	46 (82.1%)	0.015
Body mass index, kg/m2	27.2 [23.9; 31]	29.3 [26; 32.25]	0.052
Current smoker, % (n)	12 (34.3%)	13 (23.2%)	0.33
Diabetes, % (n)	8 (22.9%)	19 (33.9%)	0.35
Hypertension, % (n)	30 (85.7%)	54 (96.4%)	0.1
Degree of stenosis, %	70 [70; 80]	80 [70; 87.5]	0.052
Symptoms, % (n)	9 (25.7%)	15 (26.8%)	1

Numbers given as counts (percentage) or median [interquartile range]. Between groups comparisons were made with the help of the Mann-Whitney U test (quantitative), or with the help of accurate two-sided F-test (qualitative). P values < 0.05 were considered significant.

Atherosclerotic plaques were isolated by CEA under general anaesthesia with transcranial Doppler (TCD) monitoring, online electroencephalogram (EEG) registration and systemic heparinization. The plaques were washed with sterile 0.9% NaCl, placed in IMDM (Invitrogen, Carlsbad, CA, USA) and immediately transported to the laboratory for processing. Based on the histological evaluation and the stage of atherosclerosis plaque stability/vulnerability status was determined for each preparation according to a modified, well-defined, well-validated American Heart Association (AHA) atherosclerotic scoring system [13,14]. The main criteria for vulnerable plaque definition were a fibrous cap thickness < 65 μm, high macrophage infiltration (> 25 cells per 0.3 mm diameter field), fibrous cap rupture and/or erosion, intraplaque hemorrhages and/or thrombosis [15]. Histological samples were independently analyzed by two histopathologists (I.M. and R.D.) who were blinded to patient’s data. All ambiguously classified plaques were excluded from the study.

### Plaque inner mass cells purification and cell culture

Plaque tissue samples with average size 12±1×4±1×6±1 mm and weight 300–500 mg were treated two times with at least 2 volumes (volume : weight) of lysis buffer (0.5% NP-40, 2 mM EDTA, 0.01 M Tris-HCl, pH = 7.5) for 10 minutes at the room temperature. Then tissue samples were washed 5 times with PBS, minced finely into small pieces (1 mm^3^) with subsequent incubation in 0.2% collagenase II (Invitrogen, Carlsbad, CA, USA) in Hanks’ solution (H6648, Sigma Chemical Co., St. Louis, MO, USA) for 16 h at 37°С, 5% CO_2_. Hydrolyzed plaque fragments were centrifuged 10 min at 400 g, resuspended in 6 ml of IMDM, 10% FBS, 100 μg/ml penicillin and streptomycin (Invitrogen, Carlsbad, CA, USA) (IMDM-FBS), placed in the 10 mm tissue culture dishes and cultivated at 37°C, 5% CO_2_. After 3 days adherent PIMC were washed 3 times with IMDM to remove tissue fragments, fresh IMDM-FBS was added and cells were cultivated at 37°C, 5% CO_2_ with a medium change every 5 days for 20–25 days until about 80% confluence. Cells were subcultured with the solution of 0.2% collagenase II in IMDM.

The morphology of cells was documented on Axiovert 40 С microscope with AxiocamICc 3 camera using AxioVisionSE 64 Rel.4.9.1 software (CarlZeiss, Munich, Germany).

To investigate the proliferation and/or differentiation PIMC potential several culture media were used: DMEMF12, 15% FBS, 10 nM dexamethazone, 50 μg/mL ascorbic acid, 0.2 μg/mL L-glutamine, 6.25 ng/mL Insulin-Transferrin-Sodium Selenite (DMEM F12-chondro) [16], DMEMF12, 15% FBS, 100 nM dexamethazone, 50 μg/mL ascorbic acid, 0.2 μg/mL L-glutamine, 10 mM β-glycerophosphate (DMEMF12-osteo) [16] and Medium 231 with smooth muscle growth supplement (Invitrogen, Carlsbad, CA, USA) (Medium231s), which are used as chondrogenic, osteogenic and smooth muscle cell (SMC) growth/differentiation media, correspondingly.

Effects of proliferation-inducing agents were investigated by the addition of the following reagents to PIMC in IMDM-FBS: 10 ng/mL phorbol 12-myristate 13-acetate (PMA), 2 ng/mL insulin-like growth factor (IGF), 50 ng/mL epidermal growth factor (EGF) (Sigma-Aldrich, Saint Louis, MO, USA) or FBS to a 20% content.

Cell viability was quantitatively estimated 1 and 5 days after PIMC seeding in various media or with proliferation-inducing agents in a 48-well plate (10^4^ cells per well) using alamarBlue reagent (Invitrogen, Carlsbad, CA, USA).

### Isolation and cell culture of human umbilical vein endothelial cells (HUVEC), vascular smooth muscle cells (VSMC) and hyaline cartilage chondrocytes (HCC)

HUVEC were isolated from umbilical cord vein and cultured in IMDM-FBS up to 4 passages as previously described [17].

VSMC were isolated from a fragment of internal carotid artery, removed while surgical treatment/correction of the kinked internal carotid arteries. HCC were isolated from the hyaline cartilage of femoral head removed during total hip replacement. The tissue fragments were washed with PBS, minced finely, incubated in collagenase II, put in Petri dishes and washed after three days as described above for PIMC. VSMC and HCC were cultured in IMDM-FBS and DMEM F12-chondro, respectively, and subcultured with collagenase II.

### MiRNA isolation and RT-TaqMan PCR

MiRNA were isolated by phenol-based extraction (Trizol, Invitrogen, Carlsbad, CA, USA) from treated and untreated with lysis buffer plaque tissue samples homogenized in liquid nitrogen according to the manufacturer’s protocol. RNA samples were treated with RNase-free DNase I (Fermentas, Vilnius, Lithuania) and precipitated in 70% ethanol, 0.3 М NaAc.

Primers and probes for reverse transcription and TaqMan PCR (sequences are provided in S1 Table) were synthesized in the Laboratory of medical chemistry (ICHBFM SB RAS, Novosibirsk, Russia). Synthesis of cDNA was carried out in 20 μL by reverse transcription of 100 ng RNA using 0.05 mM RT-primer and 50 U M-MuLV reverse transcriptase (BiolabMix, Novosibirsk, Russia) according to the manufacturer’s protocol. Quantitative real-time PCRs were performed in duplicate in 24 μL and contained 2.5 μL cDNA, 0,5 μM forward primer, 0.4 μM universal reverse primer, 0.3 μM TaqMan probe, buffer BioMaster (BiolabMix, Novosibirsk, Russia). The following thermocycling condition was used for miRNA 23a and miRNA 451a: 95°C for 5 min and 40 cycles of 95°C for 15s / 60°C for 45 s.

### RNA extraction from PIMC

Total RNA was isolated from the first passage s- and v-PIMC with Trizol reagent. RNA samples were treated with RNase-free DNase I and precipitated in 70% ethanol, 0.3 М sodium acetate, 20 μg glycogen. The concentration and RNA integrity number (RIN) were determined using NanoDrop 2000 (Thermo Fisher Scientific, Wilmington, DE, USA) and Agilent RNA 6000 Pico Kit on Agilent 2100 Bioanalyzer (Agilent Technologies, Santa Clara, CA, USA).

### mRNA sequencing and data analysis

RNA-Seq was performed in the Turku Centre for Biotechnology Finnish Microarray and Sequencing Centre (http://www.btk.fi). Two RNA samples from s-PIMC and two samples from v-PIMC were prepared for the sequencing using Illumina TruSeq Stranded mRNA Sample Preparation Kit and sequenced with the HiSeq 2500 instrument (Illumina, San Diego, CA, USA) using single-end sequencing chemistry and 50bp read length. RNA-Seq reads were quality-filtered using FASTQ Quality Filter (-q 20 –p 75) from FASTX-toolkit (http://hannonlab.cshl.edu/fastx_toolkit/). Ribosomal RNA reads were filtered out using SortMeRNA tool [18]. RNA-seq reads were aligned on GRCh38 (Ensembl release 91) using HISAT2 aligner (—score-min L,0,-0.5) [19]. FeatureCounts tool from the Subread package was used to count reads to genomic features [20]. Differential expression analysis and PCA visualization were performed with DESeq2 [21]. Heatmaps were generated using R. RNA-seq data were deposited into NCBI’s Gene Expression Omnibus (GSE116243).

For comparative analysis using PCA and heatmaps the following publicly available RNA-Seq datasets were used: GSE51878 from human coronary artery smooth muscle cell (HCASMC) [22], GSE85784 from HAEC and HUVEC [23], GSE79044 from monocyte-derived macrophages (MCRPH) [24], ENCSR000CUE, ENCSR000CUF and ENCSR000CUJ (ENCODE Consortium repository) from human articular chondrocytes of knee joint (AChKJ), human osteoblasts (HOB) and human fibroblasts from aortic adventitia (FAADV), correspondingly.

### Immunofluorescence

PIMC (10^4^ cells per well) were seeded in 8-well Nunc Lab-Tek Chamber Slides (Invitrogen, Carlsbad, CA, USA). After reaching the 50% confluence cells were washed with IMDM, fixed with 3.7% paraformaldehyde in IMDM, and 0.2% Nonidet P-40 was used to permeabilize the cells. Slides were incubated for 2 hours at 37°С with primary antibodies against α-SMA (ab7817), aggrecan (ab36861), fibronectin (ab45688), СD34 (ab81289), VEGF-A (ab1316) (Abcam, Cambridge, UK), CD105 (M352701-2), CD31 (PECAM1) (M082301-2) (Dako, Agilent, Santa Clara, CA, USA), CD14 (2109035) (Sony Biotechnology Inc., San Jose, CA, USA), then incubated for 1 hour at 37°С with Alexa 488-conjugated secondary antibodies (ab150077 and ab1500117, Abcam, Cambridge, UK). Samples were stained with rhodamine phalloidin (Sigma-Aldrich, Saint Louis, MO, USA) for F-actin, and glass slides were covered with the Prolong Gold antifade reagent with DAPI (Invitrogen, Carlsbad, CA, USA) for nuclei staining, mounted with coverslips and analyzed using confocal laser scanning microscopes LSM 510 UV Meta or LSM 780 NLO (Carl Zeiss, Jena, Germany) using three laser lines at 405 nm (to detect cell nuclei stained with DAPI), 543 nm (to detect actin microfilaments stained with phalloidin-TRITC), and 488 nm (to detect Alexa Fluor 488 secondary antibodies).

### Statistical analysis

Data are presented as mean ± SD or median with range. Where appropriate data were normalized using log_2_ and statistical comparisons were made using T-tests. Where normality could not be confirmed, data were analysed using a Mann-Whitney test. P values < 0.05 was considered statistically significant. Benjamini-Hochberg FDR adjustment was applied to correct for multiple comparisons in DEG identification.

## Results

### The removal of cells from the surface of plaques

To eliminate cells located on the atherosclerotic plaque surface or close, we suggest a short-term plaque treatment with lysis buffer containing NP-40 detergent. NP-40 concentrations above 0.5% provide efficient lysis of cells and can guarantee the elimination of plaque surface cells. At the same time, due to detergent micellization, dense plaque structure and short treatment time the detergent should not be able to infiltrate deep into the plaque body, ensuring the preservation of PIMC.

The removal of cells from the plaque surface was assessed using quantitative TaqMan PCR for miR-451а and miR-23a. While miR-451a is highly expressed in blood cells, whereas miR-23a is expressed at a low level in various cell types [25]. Our data showed that a large portion of surface plaque cells is comprised of red blood cells, and some of the plaques appear visibly “bloody” after surgical removal (S2 Table). The decrease of ΔCt = Ct(miR-23a)-Ct(miR-451a) after lysis buffer treatment indicates the decrease in the number of blood cells in the sample. Ten plaques (6 vulnerable and 4 stable) were divided into two parts, miRNA levels were measured with and without lysis buffer treatment and the decrease of ΔCt was observed in all samples treated with lysis buffer as compared to controls (Table 2, P = 0.0047).

**Table 2:** The relative expression of miR-451a and miR-23a (ΔCt = Ct(miR-23a)-Ct(miR-451)) in v-plaque samples (v) and s-plaque samples (s).

Plaque	ΔCt (control)	ΔCt (lysis buffer)
v1	2.2	1.6
v2	6.7	3.1
v3	7.1	4.2
v4	5.3	3.8
v5	8.2	4.9
v6	4.4	3.2
s1	2.8	2.3
s2	4.8	4.7
s3	4.4	3.0
s4	1.6	1.4

MicroRNA level was measured after the isolation from untreated (control) and treated with lysis buffer (lysis buffer) plaque fragments. The ΔCt decrease (Paired samples two-tailed t-test P = 0.0047) was observed after the treatment in all plaque samples indicating a reduction in blood cell quantity.

The raw Ct data (S2 Table) demonstrate that the miR-23a level did not change significantly after lysis buffer treatment (P = 0.4417) and the ΔCt decrease was caused by the increase of miR-451а Ct (P = 0.0029) after lysis buffer treatment. Using miRNA expression as an indicator in preliminary experiments, we optimized the detergent concentration in lysis buffer and treatment time to achieve the maximum decrease in ΔCt.

**Fig 1:**
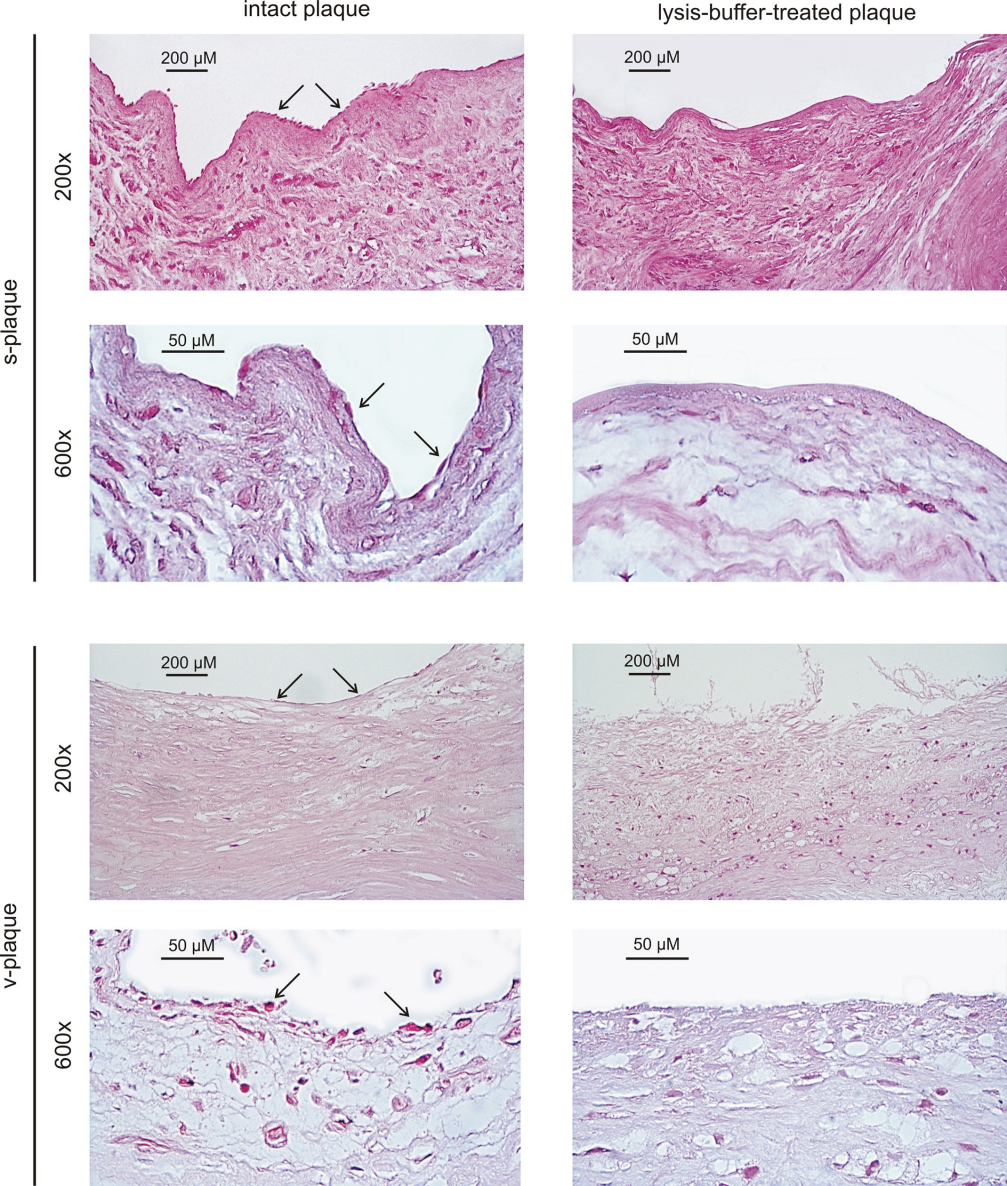
Lysis-buffer-treated versus intact plaques. H&E staining of s- and v-plaques on shows elimination of plaque surface cells (arrows) after lysis-buffer treatment.

It should be noted, that PIMC may also express both miRNAs, and therefore the decrease of ΔCt is a helpful indicator but should be confirmed by other means. To visually assess the presence of cells on plaque surface we have compared histological specimens obtained from the same plaque before and after lysis buffer treatment (Fig 1). Both for stable and vulnerable atheromas, lysis buffer successfully removed plaque surface cells (including endothelial cells), without any noticeable damage to PIMC. Based on the observations provided by both methods all plaques studied further were treated with lysis buffer and thoroughly washed with PBS prior to PIMC isolation.

### PIMC purification and cell culture

In the preliminary experiments lysis buffer-treated, washed and finely minced plaques were incubated with proteases (dispase, collagenases type I, type II and type IV), commonly used for isolation of cells from various tissues [7,26]. According to our results most of the enzymes as well as short (2–4 hours) enzymatic treatment of minced plaque tissue did not result in noticeable PIMC release. We have therefore developed the protocol for PIMC isolation based on our previous successful experience with the isolation of chondrocytes from human cartilage–a tissue of comparable density. As such, we used long-term collagenase II treatment, which is common for chondrocyte isolation from cartilage [27]. We have observed that long-term overnight treatment of minced plaques with 0.2% collagenase II has efficiently hydrolyzed the dense plaque connective tissue without noticeable influence on PIMC viability and subsequently this protocol was used to isolate PIMC.

**Fig 2:**
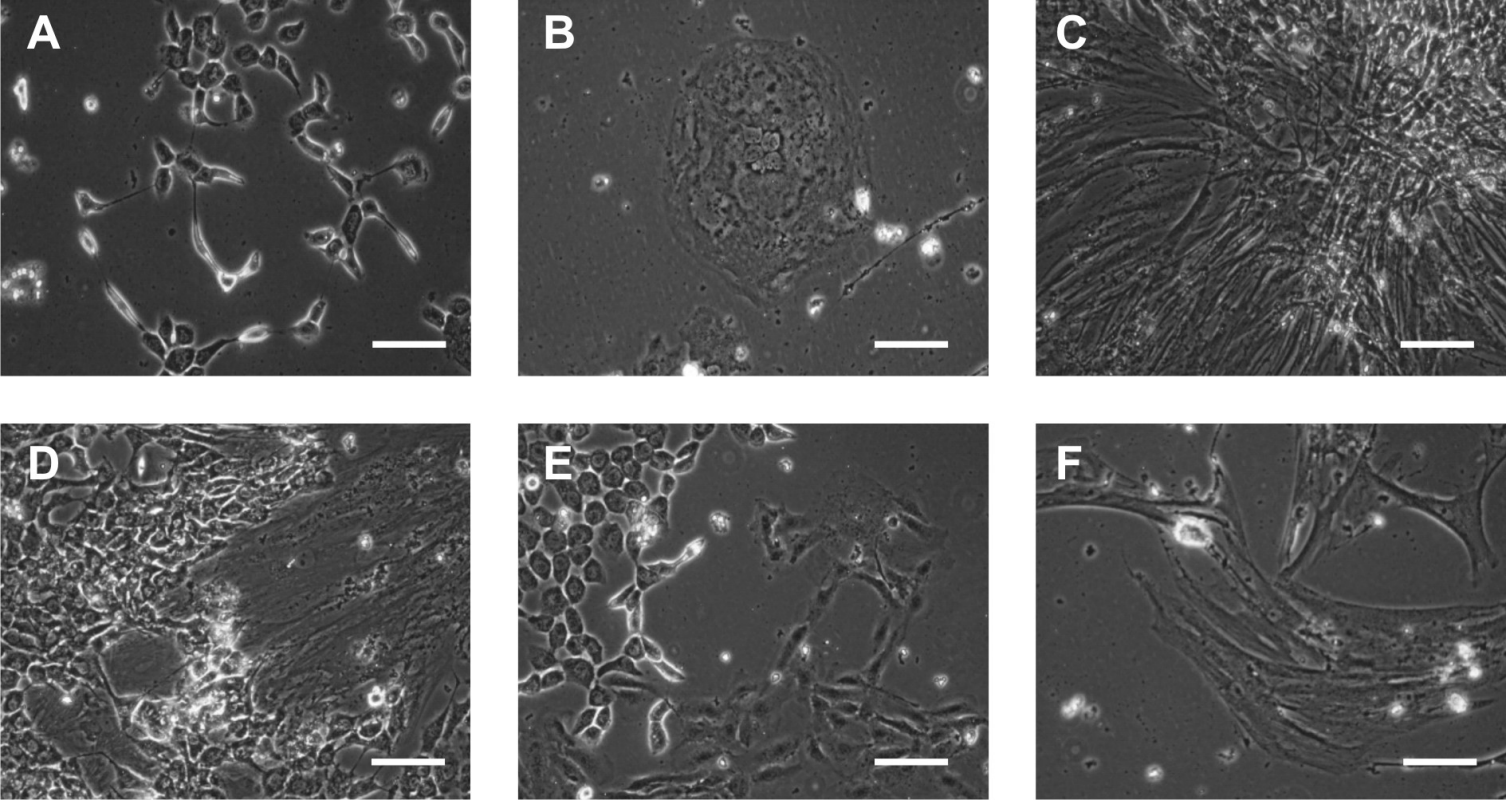
PIMC morphology. Cells from v-plaque at passage 0 (cultured for 15 days). Phase contrast images representative of the 48 obtained PIMC cultures. Scale bars represent 40 μm.

The clonal growth of PIMC was observed approximately 10–15 days after seeding in culture dishes. At this stage, PIMC cultures often contained several morphological types of cells (Fig 2). PIMC differed in shape, size, type of cell-to-cell contacts and the colony pattern. One type of cells with well-defined borders formed branched strands (Fig 2A), while other cells grew in multilayered colonies (Fig 2B–2E). The colonies of large elongated (80±20 × 20±10 μm) cells with numerous contacts were frequently found in PIMC cultures (Fig 2F). All described cell morphotypes proliferated, but after subculturing cell cultures became more homogenous. It should be noted, that not all cultured PIMC were characterized by the presence of multiple cell morphotypes and large cells (Fig 2F) were the predominant type of cells in most of the plaque cultures.

We managed to isolate PIMC from 30 out of 35 s-plaques and 35 out of 56 v-plaques. Among them PIMC from only 28 s-and 20 v-plaques proliferate *in vitro* (Table 3). Apparently, some plaques did not yield enough cells which could be cultured, especially vulnerable plaques, which are known to have extensive cell death regions (necrotic cores) [1].

**Table 3:** The efficacy of PIMC isolation and culture from stable (n = 35) and vulnerable (n = 56) plaques.

	Stable plaques (n = 35)	Vulnerable plaques (n = 56)
Successful PIMC isolation	30 (86 %)	35 (62 %)
PIMC cultured at least 2 passages	28 (80 %)	20 (36 %)

We have compared the efficacy of s-and v-PIMC isolation, simultaneously extracting cells from plaque tissue sample of similar weight (400 ± 30 mg) and estimating the quantity of cells after 20-days culturing. The density of s-PIMC in culture dishes was about 10^4^ per cm^2^ versus 2×10^3^ per cm^2^ for v-PIMC. PIMC from s- and v-plaques doubled in 5±2 days and 8±2 days, respectively, thus indicating that cells from stable plaques proliferate faster. PIMC remained viable for 40–45 days while being subcultured 2–3 times. Further cultivation resulted in cell detachment from culture dishes and significant amount of cell death was observed. In this study, cells on the first passage were used in all experiments.

**Fig 3:**
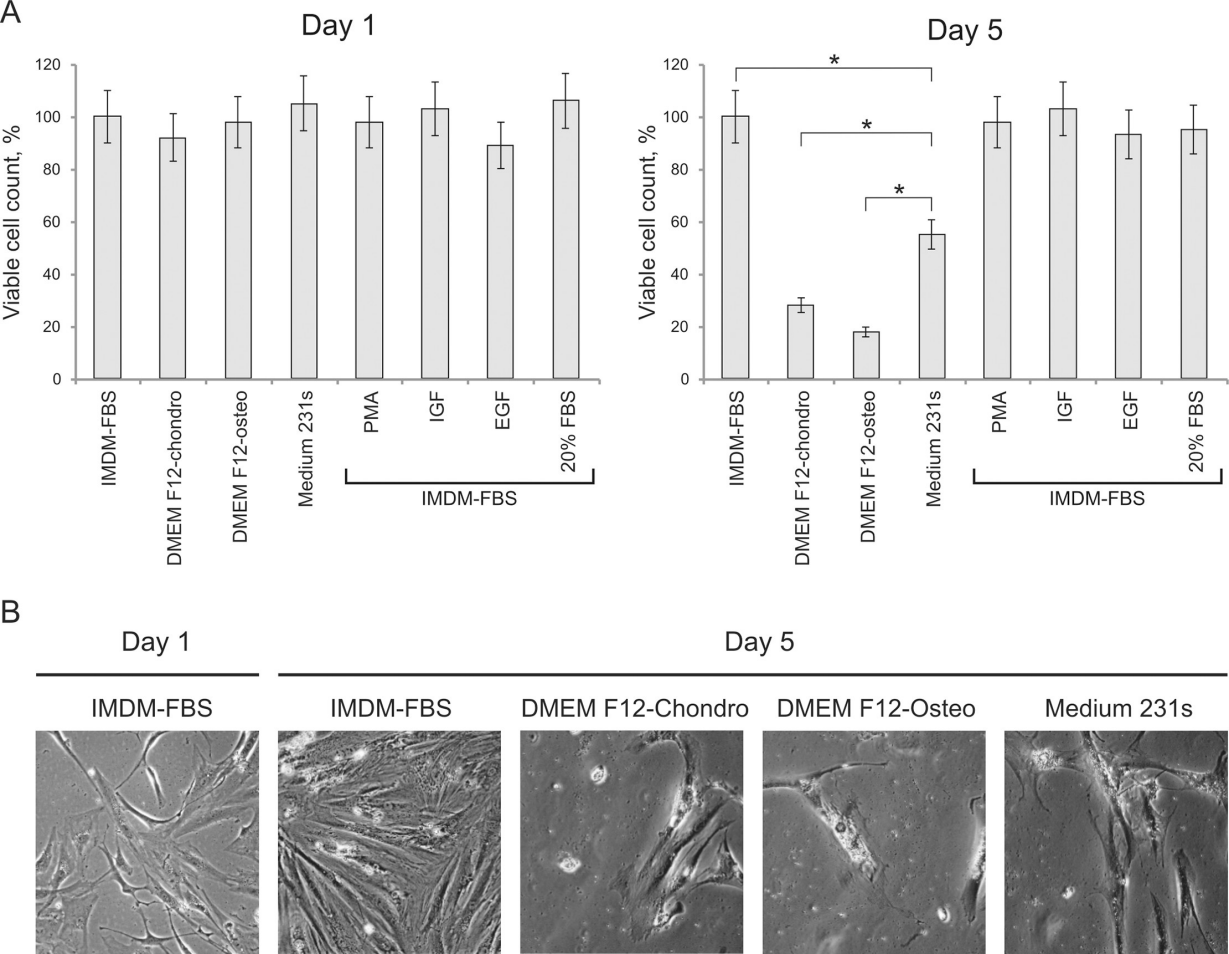
PIMC in various culturing conditions. A. s-PIMC viability on the next day after subculturing (Day 1) and after 5-day incubation (Day 5) in various culture media or with different growth stimulators normalized to viability of s-PIMC in IMDM-FBS (first column of the corresponding panel). Mean of three replicates with standard deviation. Asterisk (*) indicates a significant difference in cell viability (P < 0.0001, T-test). B. Phase-contrast microscopy images of s-PIMC on Day 1 and Day 5. Representative images from two independent experiments are shown. Scale bars represent 20 μm.

We tested several different culture media compositions for PIMC culturing. Given the presence of calcification in atherosclerotic plaques and the potential SMC origin of PIMC, we tested if PIMC would grow on chondrogenic/osteogenic culture media or Medium 231s, designed for SMC culture. In Medium 231s cells were viable, but did not proliferate as compared to IMDM-FBS, where cells doubled after a 5-day incubation. In IMDM-chondro and IMDM-osteo cells were even less viable, than in Medium 231s (Fig 3A and 3B), suggesting a possible cell death. Addition of extra FBS (up to 20%) or proliferation-inducing agents (PMA, IGF-1, EGF) did not increase PIMC proliferation rate in IMDM-FBS (Fig 3A). Presumably, PIMC culturing in specific media induces terminal differentiation and apoptosis, however, from our results we can only conclude that PIMC are sufficiently different from the cells, which are normally cultured in these media. As such, IMDM-FBS was the optimal medium for PIMC culture from the three tested media.

### Gene expression profiling of PIMC

We performed high-throughput sequencing of PIMC polyA-enriched transcriptome, isolated from two stable and two vulnerable atherosclerotic plaques. For each sample we obtained up to 15 million reads (S3 Table), of which over 95% mapped to the human genome (GRCh38 Ensembl release 91). GC content of all samples followed a normal distribution with expected peaks at 40–60%. To perform a general comparison of gene expression profiles of s- and v-PIMC with other cell types we used publicly available RNA-seq datasets of cells involved in atherogenesis: smooth muscle cells (HCASMC), monocyte-derived macrophages (MCRPH), vascular endothelial cells (HUVEC and HAEC), adventitia fibroblasts (FAADV), chondrocytes (AChKJ) and osteoblasts (HOB).

**Fig 4:**
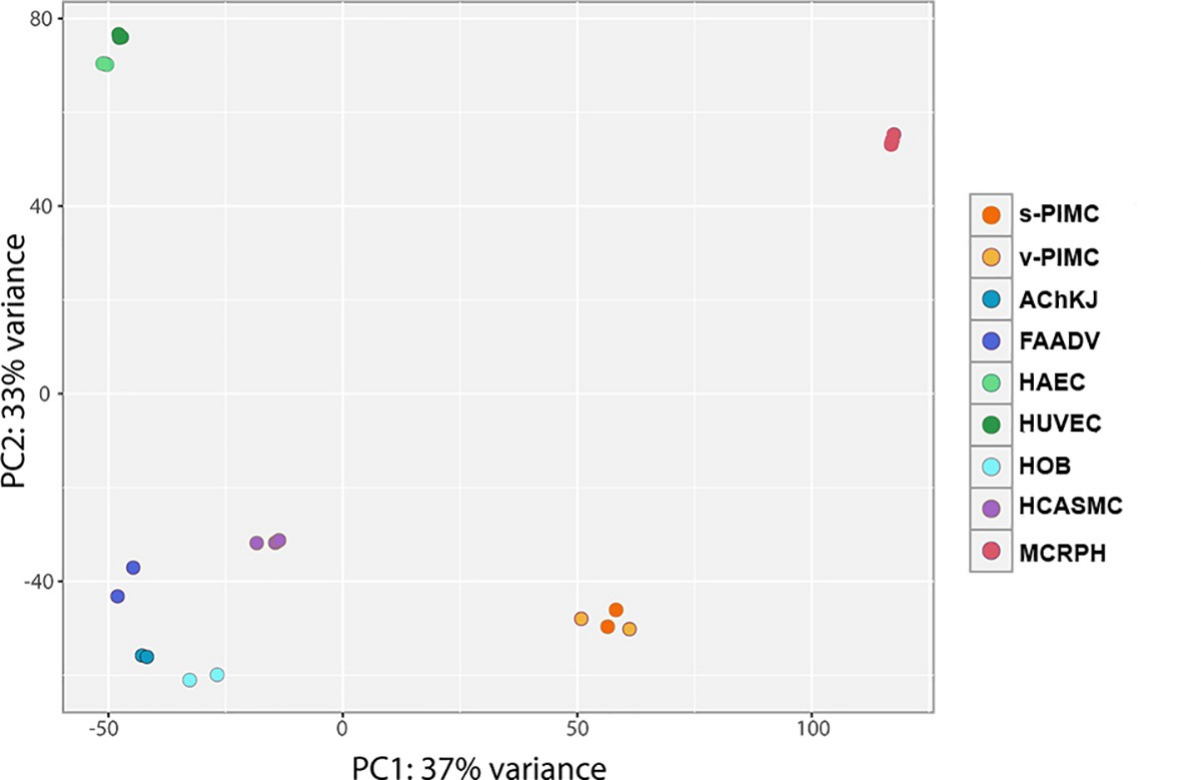
Principal component analysis of gene expression profiles. RNA-Seq gene expression data of v-PIMC (n = 2), s-PIMC (n = 2) and cells involved in atherogenesis (vascular smooth muscle cells–HCASMC (n = 3), endotheliocytes–HUVEC (n = 3) and HAEC (n = 3), macrophages MCRPH (n = 3), chondrocytes–AchKJ (n = 2), osteoblasts HOB (n = 2) and fibroblasts–FAADV (n = 2)). PCA demonstrates the major components (PC1 and PC2) of the variance to separate PIMC from other cells.

**Fig 5:**
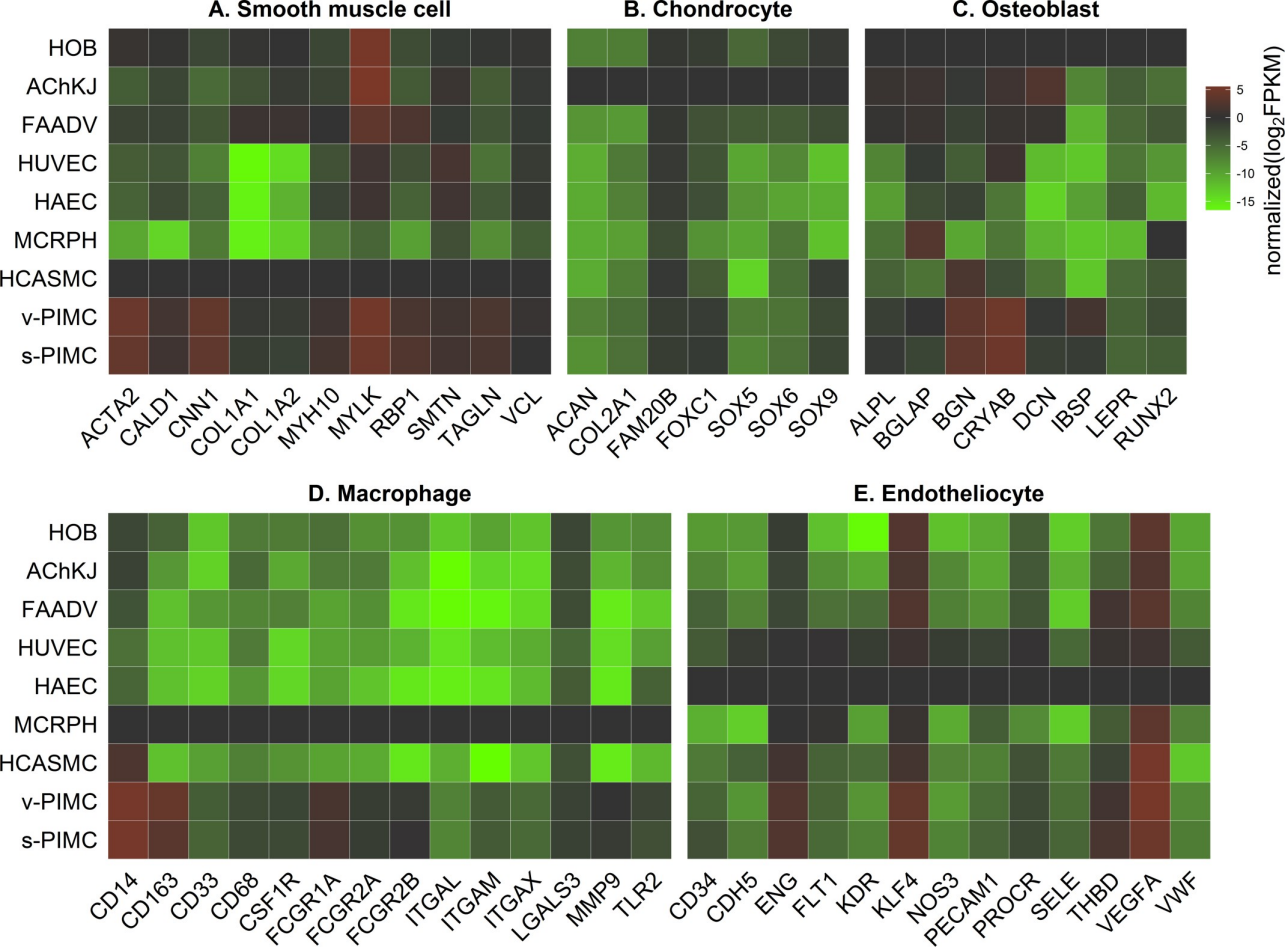
Heatmaps of cell type-specific gene expression. RNA-Seq gene expression data of v-PIMC (n = 2), s-PIMC (n = 2) and cells involved in atherogenesis (vascular smooth muscle cells–HCASMC (n = 3), endotheliocytes–HUVEC (n = 3) and HAEC (n = 3), macrophages MCRPH (n = 3), chondrocytes–AchKJ (n = 2), osteoblasts HOB (n = 2) and fibroblasts–FAADV (n = 2)). Panels A-E show the expression of cell-type specific markers. Expression of each marker is normalized to the expression level in the corresponding specific cell type. Red: upregulation; green: downregulation.

The principal component analysis (PCA) demonstrated major differences between PIMC and other cell types. Connective tissue cells (fibroblasts, VSMC, chondrocytes and osteoblasts) were clustered together; PIMC, endothelial cells and macrophages all formed separate well-defined groups (Fig 4). Furthermore, heatmaps showing the relative mRNA expression of key markers of various atherosclerosis-related cell types (smooth muscle-, endothelial, macrophage, chondrocyte and osteoblast) display the unique PIMC expression pattern of these proteins (Fig 5). Most of VSMC markers were highly expressed in PIMC - 2–40 fold higher FPKM then in control primary VSMC. One exception was the collagen I gene (*Col1A1*), which was expressed at a 50% FPKM level (Fig 5A). Macrophage markers were also expressed in PIMC, although their pattern was different from the control cultured macrophages, probably due to great flexibility and plasticity of both cell types depending on signals in their environments (Fig 5D) [24]. PIMC expressed several endothelial markers, however, RPKM was lower than in HUVEC and HAEC cells (Fig 5E), probably due to elimination of plaque surface endothelial cells with lysis buffer (Fig 1). Judging by the expression of cell-specific markers PIMC differ from chondrocytes, but are closely related to osteoblasts (Fig 5B and 5C).

We identified eight genes that were significantly differentially expressed (DEGs) in s-PIMC compared to v-PIMC at an FDR < 0.05 (Table 4). The number of DEGs was relatively low, possibly due to the low number of analyzed plaque samples, however, most of the identified genes are involved in atherogenesis, suggesting PIMC cultures as an suitable tool for investigation of the mechanisms of atherosclerosis and search of plaque vulnerability markers.

**Table 4:** Differentially expressed genes in v-PIMC (v) compared to s-PIMC (s).

**Downregula-ted genes**	**Protein coded**	**Gene function**	**Fold change (v/s)**	**P value**	**FDR**
MMP1	matrix metallopeptidase 1	Metalloprorease specific to Collagens type I, II, III	0,195	8,94E-19	1,55E-14
NPY1R	neuropeptide Y receptor Y1	G-protein-coupled receptor superfamily, receptor of NPY	0,282	5,61E-10	4,86E-06
**Upregulatedgenes**	**Proteincoded**	**Genefunction**	**Expression fold change (v/s)**	**P value**	**FDR**
PSG4	pregnancy specific beta-1-glycoprotein 4	Innate immunity (?)	2,951	1,85E-07	0,00064
IL1B	interleukin 1 beta	Pro-inflammatory cytokine	2,907	3,93E-07	0,001
CYBB	cytochrome b-245 beta chain (NOX2)	superoxide generating enzyme	2,232	1,16E-05	0,029
DSG2	desmoglein 2	cell adhesion	2,452	2,26E-05	0,044
IBSP	integrin binding sialoprotein	structural protein of the bone matrix	2,375	3,05E-05	0,048
CPNE7	copine 7	calcium-dependent membrane-binding protein	2,350	2,99E-05	0,048

### PIMC immunophenotyping

We have analysed the expression of smooth muscle cells marker α-SMA, macrophage marker CD14, extracellular matrix proteins aggrecan, fibronectin, neovascularisation markers VEGF-A, CD105, cellular adhesion receptor CD31 and progenitor/dedifferentiation receptor CD34 in three s-PIMC and three v-PIMC samples using immunofluorescence staining. Despite PIMC heterogeneity, the expression of selected markers was observed in about 80% of cells, and the patterns were similar for PIMC from different patients. The dense granules with nonspecific fluorescence observed in some PIMC were excluded from IF analysis.

**Fig 6:**
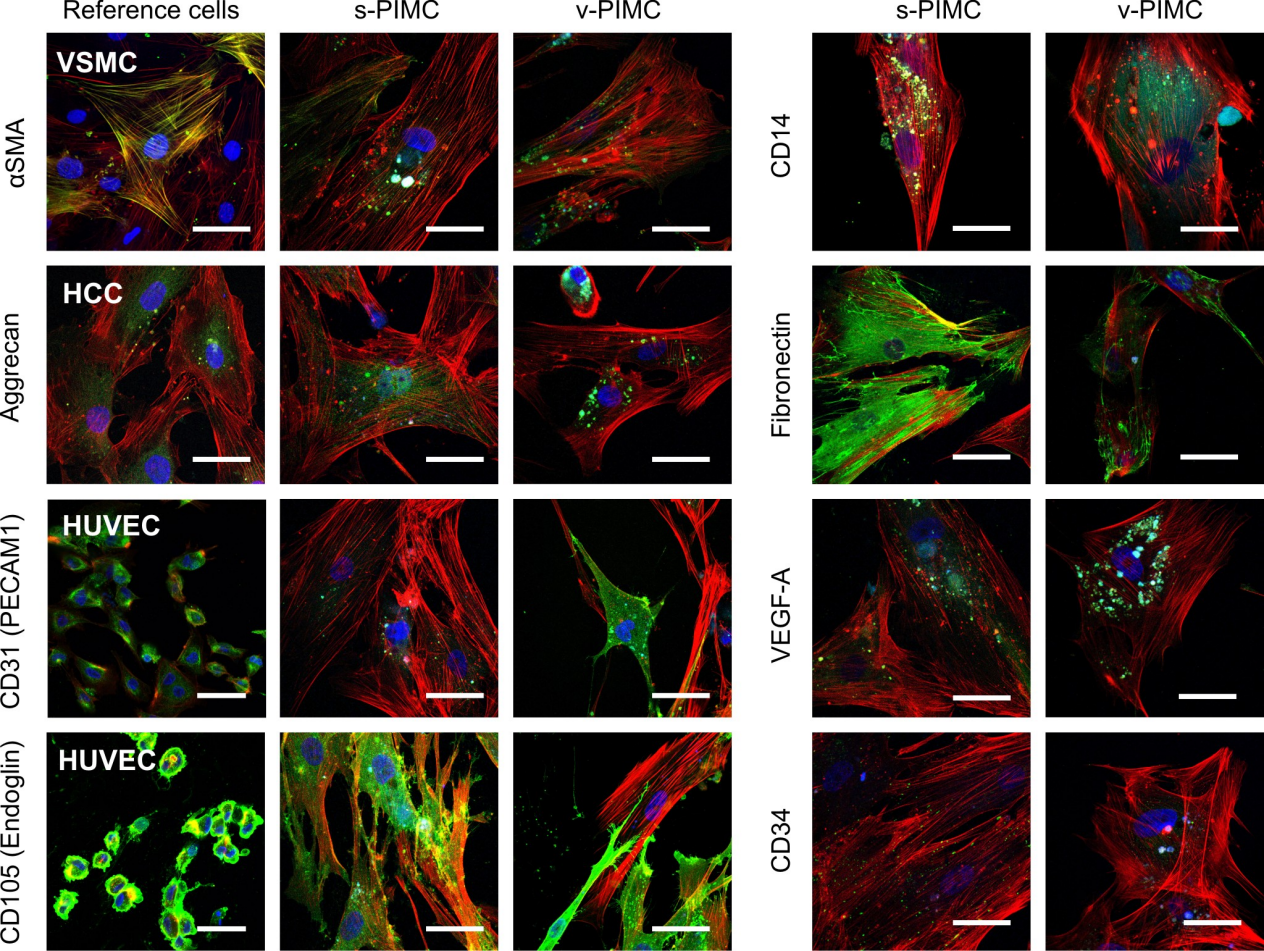
Immunofluorescence staining of s- and v-PIMC. Specific proteins are stained green (AlexaFluor 488), f-actin and nuclei are stained red and blue, correspondingly. In the example column VSMC, HCC and HUVEC are stained for indicated markers. Representative images were selected from three independent experiments performed using PIMC obtained from different donors are shown. Scale bars represent 50 μm.

Staining revealed expression pattern of several SMC, macrophage, chondrocyte and endotheliocyte markers in PIMC from s- and v-plaques, and allowed for a comparison with VSMC, HCC and HUVEC (Fig 6). The expression of SMC marker α-SMA was detected in both types of plaque cells, but cells from s-plaques expressed more α-SMA Monocyte/macrophage marker CD14 was expressed in PIMC and localized mainly in the cytoplasm.

Plaque cells expressed chondrocyte marker aggrecan, and its localization in cytoplasm and intracellular vesicles was similar to hyaline cartilage chondrocytes. Another ECM protein fibronectin was also expressed in PIMC, especially those isolated from stable plaques.

The expression of CD34, VEGF-A, CD31 (PECAM1) and CD105 (endoglin) was also observed in both types of PIMC. The expression of CD34, VEGF-A was higher in s-PIMC, whereas CD31 expression was higher and more conventionally localized in v-PIMC. CD105 was highly expressed in all PIMC, and its localization was similar to HUVEC.

## Discussion

Various cell types are considered to be involved in atherosclerotic plaque development [28]. Bonnano et al characterized cell populations in human carotid plaques using flow cytometry stating that approximately half of the cells are inflammatory mononuclear cells (26.16 ± 14.2% lymphocytes; 17.34 ± 12.6% monocytes/macrophages) and another half are SMC (56.50 ± 20.3%) [7]. Furthermore, osteoblast- and osteoclast-like cells have been identified near areas of plaque mineral deposits [29].

The data on the plaque cell populations, dense plaque structure and its slow growth suggest that relatively low number of cells is responsible for producing plaque connective tissue components. We suggest a new approach to isolation of cells entombed in the plaque connective tissue - plaque inner mass cells (PIMC) - and suggest their role as the key cell determinants of plaque structure. These cells are undoubtedly influenced by outer stimuli, but the response is modulated by the PIMC own phenotype as well as the density and structure of the surrounding connective tissue. Thus, PIMC may prove useful in exploring the response of plaque cells to external signals and assist in the study of the general mechanisms of growth and functioning of atherosclerotic lesions.

In this study, we have isolated and cultured atherosclerotic plaque inner mass cells using an optimized protocol for lysis of plaque surface cells, followed by hydrolysis of plaque tissue with subsequent inner mass cell isolation and selection for conditions for efficient PIMC cultivation. As we have expected, the quantity of these cells and their proliferative activity *in vitro* is relatively low: the doubling time is about 5 and 8 days for cells from stable and vulnerable plaques, respectively, as compared to approximately two days for VSMC from healthy aorta [30]. Attempts to improve proliferation of PIMC cells with either the culture media variations, addition of growth factors or FBS concentration increase were not successful. Presumably, *in vivo* plaque cells’ proliferation is also limited by the constrained diffusion in the dense plaque tissue, lack of the nutrition and necessary growth factors. Advanced atherosclerotic plaques can grow for years before removal by CEA [31] and therefore it’s no wonder that isolated PIMC proliferate slowly. Our data showed that PIMC could contain several morphological types of cells after isolation, but during passaging cell culture become more homogenous. It is not clear if this phenomenon is concerned with the overgrowth of cells with higher proliferative activity or *in vitro* dedifferentiation of PIMC due to their plasticity.

High-throughput mRNA sequencing of s- and v-PIMC demonstrated that PIMC have a unique gene expression profile. The principal component analysis showed that plaque cells do not group with any of cells involved in atherogenesis, however, all four PIMC cultures group together, indicating overall reproducibility of PIMC extraction protocol. Moreover, PIMC express mRNAs encoding for markers of atherosclerosis, as well as markers of macrophages, VSMC, endotheliocytes, chondrocytes and osteoblasts. The complicated gene expression profile of PIMC could be attributed to their heterogeneity and/or plasticity of plaque cells, since during atherogenesis cells may lose or acquire certain markers [32].

The RNA-Seq data on the expression of both SMC and macrophage markers in PIMC are supported with the positive immunofluorescence staining of the cultured plaque cells for SMC marker α-SMA (*Acta 2*) andmonocyte/macrophage marker CD14. It is known that CD14+ monocytes and macrophages are involved in plaque inflammation and plaque structure remodeling [33]. Until recently, it was generally assumed that intimal proliferative or synthetic smooth muscle cells originate from medial contractile smooth muscle cells, while macrophages differentiate from monocytes that transmigrate through the endothelial layer upon vascular injury [33,34]. However, recent lineage tracing studies have shown that dividing plaque cells into macrophages and SMC based on the traditional markers’ expression is not accurate: during atherogenesis SMC acquire pro-inflammatory phenotype, losing the expression of SMC markers and gaining macrophage markers [35]. Regulation of SMC phenotype switching depends on the location of cells in the plaque and, apparently, is regulated through the stem cell and induced pluripotent stem cell factor KLF4 [36]. The opposite is also possible: plaque macrophages (or at least cells of hematopoietic origin) may transdifferentiate into ECM-producing cells and express SMC markers, particularly α-SMA [32]. The decrease in transdifferentiated macrophages and increase in pro-inflammatory M1 macrophages, as well as SMC transdifferentiation to a macrophage phenotype lead to the plaque vulnerability [32,35].

Along with SMC-to-macrophage transdifferentiation, the potential of SMC to acquire the phenotype of chondrocyte-like cells has been reported [37]. Moreover, myeloid calcifying cells (monocytes expressing osteocalcin and bone alkaline phosphatase) promote atherosclerotic calcification [38]. The presence of calcification in atherosclerotic plaques suggests the presence of cells similar to chondrocytes, osteocytes and even osteoclasts [29]. In PIMC we have also observed the mRNA expression of certain chondrocyte and osteoblast markers, which was also confirmed by immunofluorescence staining for aggrecan. For years, a debate revolved around whether calcification is a marker of plaque stability. Current consensus is that strong calcification indicates a highly vulnerable patient rather than a vulnerable plaque [6].

PIMC highly express ECM and adhesion protein fibronectin, which role in atherogenesis is rather controversial. It has been shown that fibronectin deposition at the sites of early plaque formation promotes atherosclerosis, but also promotes the formation of the protective fibrous cap [39,40].

PIMC express such proteins as CD34, CD31, VEGF-A and large amounts of CD105. СD34 is considered as the common progenitor and dedifferentiation marker and is expressed in various cell types, including mesenchymal stromal cells, interstitial cells and vascular endothelial cells [41]. CD31 (PECAM1) receptor is expressed exclusively by the blood vessel interfacing cells (leukocytes, platelets and endotheliocytes) and plays a major role in vascular homoeostasis: adhesion, angiogenesis, activation of T-cells and platelets. This receptor has several isoforms which may have inverse functions. For instance, normally CD31 acts to maintain endothelial integrity, however, the pro-inflammatory cytokines promote the loss of the its domain responsible for cell contacts, resulting in increased vascular permeability [42]. Moreover, the loss of inhibitory peptide in CD31 structure leads to the increased T-cell activation and migration to atherosclerotic lesion [43].

Angiogenesis markers VEGF-A and CD105 are mainly expressed in endothelial cells, though in atherosclerotic lesions they were also detected in macrophages and partially differentiated VSMC [44,45]. Neovascularization affects the atherosclerosis progression providing plaque with oxygen and lipoproteins, promoting plaque growth and leukocyte infiltration. Immaturity of neo-vessels also leads to intra-plaque hemorrhages, which are among the key factors for plaque vulnerability [5].

The new evidence about the nature of plaque vulnerability and novel non-invasive methods to determine plaque type are urgently needed. While in symptomatic carotid occlusions CEA is obligatory, in asymptomatic lesions surgery may not be required [46]. We have identified ten differentially expressed genes (FDR ≤ 0.05) in v-PIMC vs s-PIMC, most of which are related to atherogenesis. Our data on matrix metallopeptidase 1 (MMP1) (collagenase which preferentially cleaves type III collagen [47]) hypoexpression in v-PIMC versus s-PIMC support previous reports on MMP-1 from transgenic apolipoprotein E knockout mice [48]. Although metalloproteinases are believed to contribute to plaque expansion and rupture [47], our data suggest that remodeling of the plaque ECM by MMP-1 is beneficial in the progression of the lesions.

The pro-atherogenic roles of the bone sialoprotein (IBSP), superoxide generating enzyme NOX2 and the inflammatory cytokine IL-1β have been previously described [49–51]. The effects of these proteins are consonant since oxidative stress in atherosclerotic lesions can induce IBSP expression via oxidized low-density lipoproteins (oxLDLs) [52] and promote inflammation through redox-sensitive transcription factors (e.g., NF-κB and AP-1) [50]. While the involvement of IL-1β in plaque vulnerability has already been established [51], the contribution of NOX-2 to plaque rupture remains uncertain [50]. Our data on the elevated expression of IBSP, IL-1β and NOX2 in v-PIMC compared to s-PIMC support the hypothesis viewing these proteins as plaque vulnerability markers.

The differential expression of neuropeptide Y receptor Y1 (NPY1R) may account for the slower growth of v-PIMC versus s-PIMC since activation of Y1 receptor stimulates mitogenesis in VSMC [53]. The involvement of NPY and its receptors in atherogenesis and the decrease in expression of NPY1R mRNA in plaque SMCs compared to control healthy SMCs have been shown recently [10].

Little is known about copine7 (CPNE7), pregnancy-specific glycoprotein 4 (PSG4) and desmoglein2 (DSG2) proteins and to our knowledge there is no evidence of their involvement in the atherosclerosis progression. However, their involvement in atherosclerosis-related processes, such as cellular adhesion, neo-angiogenesis (DSG2, PSGs) [54,55] and innate immunity (PSGs, CPNE7) [55,56] suggests these proteins as potential atherosclerosis markers.

## Conclusion

Despite the fact that cultured cells may differ from their original *in vivo* phenotype, they can maintain the expression of crucial markers and their interrogation may inform our knowledge of the original tissue. Cultured plaque cells may be used to search for the new markers of plaque vulnerability, as well as the factors defining and changing the phenotype of plaque cells. Moreover, PIMC may be a good model for *in vitro* modulation of plaque cell death, regulation of interleukin and adhesion molecule expression and testing of anti-atherosclerosis drugs.

## Supporting information

S1 Table Primer sequences. (PDF)

S2 Table The expression of miRNA in v-plaque samples (v) and s-plaque samples (s) before and after treatment with lysis buffer. (PDF)

S3 Table RNA-seq data characteristics. (PDF)
