# Supplementary material for: Isolation, culturing and gene expression profiling of inner mass cells from stable and vulnerable carotid atherosclerotic plaques
Source: PLoS One. 2019 Jun 26;14(6):e0218892. doi: 10.1371/journal.pone.0218892 (PMC6594632; doi:10.1371/journal.pone.0218892)
Supplement: S1 Table — (PDF) [file pone.0218892.s001.pdf]

S1 Table. Primer sequences.

Universal reverse primer	5’-GTGCAGGGTCCGAGGT-3’
**miRNA 23a**	RT: 5’-GTCGTATCCAGTGCAGGGTCCGAGGTATTCGCACTGGATACGACGGAAAT-3’<br>Forward: 5’-CTATCACATTGCCAGGGA-3’<br>Probe: 5’-(FAM)-TCGCACTGGATACGACGGAAAT-(BHQ1)-3’
**miRNA 451a**	RT: 5’-TCGTATCCAGTGCAGGGTCCGAGGTATTCGCACTGGATACGACAACTCA-3’<br>Forward: 5’-CTGCAAACCGTTACCATTACT-3’<br>Probe: 5’-(FAM)-TCGCACTGGATACGACAACTCA-(BHQ1)-3’
