# Supplementary material for: Isolation, culturing and gene expression profiling of inner mass cells from stable and vulnerable carotid atherosclerotic plaques
Source: PLoS One. 2019 Jun 26;14(6):e0218892. doi: 10.1371/journal.pone.0218892 (PMC6594632; doi:10.1371/journal.pone.0218892)
Supplement: S2 Table — (PDF) [file pone.0218892.s002.pdf]

S2 Table. The expression of miRNA in v-plaque samples (v) and s-plaque samples (s) before and after treatment with lysis buffer.

Plaque	Lysis buffer treatment	Ct miR-23a	Ct miR-451a	ΔCt	Photo
v1	-	24.3	22.1	2.2	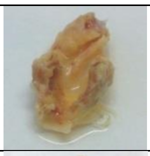
	+	24.2	22.6	1.6	
v2	-	25.8	19.1	6.7	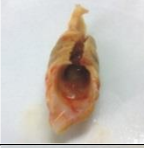
	+	25.1	22.0	3.1	
v3	-	26.9	19.8	7.1	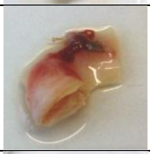
	+	25.9	21.7	4.2	
v4	-	24.1	18.8	5.3	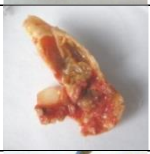
	+	25,1	21.3	3.8	
v5	-	28.5	20.3	8.2	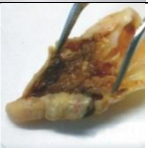
	+	26.6	21.7	4.9	
v6	-	24.1	19.7	4.4	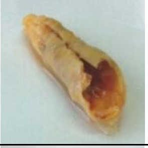
	+	24.2	21.0	3.2	
s1	-	24.0	21.2	2.8	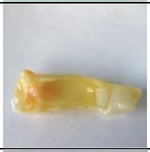
	+	25.1	22.8	2.3	
s2	-	24.8	20.0	4.8	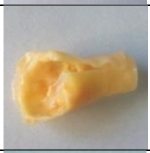
	+	24.2	19.5	4.7	
s3	-	24.4	20.0	4.4	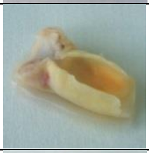
	+	23.6	20.6	3.0	
s4	-	25.3	23.7	1.6	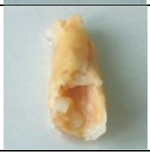
	+	25.8	24.4	1.4	

MicroRNA level was measured after isolation from untreated (-) and treated with lysis buffer (+) plaque fragments. No significant difference in Ct miR-23a after lysis buffer treatment was observed (Paired samples two-tailed t-test P = 0.4417) and the ΔCt decrease (Paired samples two-tailed t-test P = 0.0047) was caused by the increase of miR-451a Ct (Paired samples two-tailed t-test P = 0.0029).
