# Supplementary material for: Isolation, culturing and gene expression profiling of inner mass cells from stable and vulnerable carotid atherosclerotic plaques
Source: PLoS One. 2019 Jun 26;14(6):e0218892. doi: 10.1371/journal.pone.0218892 (PMC6594632; doi:10.1371/journal.pone.0218892)
Supplement: S3 Table — (PDF) [file pone.0218892.s003.pdf]

S3 Table. RNA-seq data characteristics.

Sample	Total reads	Overall alignment rate
s-PIMC_Rep1	15064453	96.925
s-PIMC_Rep2	10833985	96.545
v-PIMC_Rep1	10000494	97.075
v-PIMC_Rep2	10067280	96.85
